# Atypical chorioretinal lesions in Siberian Husky dogs with primary angle-closure glaucoma: a case series

**DOI:** 10.1186/s12917-022-03259-8

**Published:** 2022-05-16

**Authors:** Shin Ae Park, Dodd Sledge, Colleen F. Monahan, Leandro Teixeira, Ryan Boyd, Katie Freeman, Kristin Koehl, Christine Harman, Kirk Munoz, Laurence M. Occelli, Chris G. Pirie, Harriet Davidson, Simon Petersen-Jones, András M. Komáromy

**Affiliations:** 1 Department of Veterinary Clinical Sciences, College of Veterinary Medicine, Purdue University, 625 Harrison Street, West Lafayette, IN 47907 USA; 2 Department of Small Animal Clinical Sciences, College of Veterinary Medicine, Michigan State University, East Lansing, Michigan USA; 3 Michigan State University Diagnostic Laboratory, East Lansing, Michigan USA; 4 Comparative Ocular Pathology Laboratory of Wisconsin, Department of Pathobiological Sciences, School of Veterinary Medicine, University of Wisconsin-Madison, Madison, WI USA; 5 Department of Clinical Sciences, College of Veterinary Medicine, Colorado State University, Veterinary Teaching Hospital, Fort Collins, CO USA

## Abstract

**Background:**

A number of etiologies for different canine chorioretinal lesions have been proved or suggested but some fundic lesions remain unclear in terms of an etiologic diagnosis, treatment options and prognosis. The purpose of this case series is to describe atypical chorioretinal lesions observed in dogs with primary angle-closure glaucoma (PACG).

**Case presentation:**

Two spayed-female Siberian Huskies (3- and 4-year-old) and one Siberian Husky/Australian Shepherd mixed breed dog (11-month-old) that had multifocal depigmented retinal lesions and PACG were included. Procedures: Ophthalmic examination, gross, and histopathologic examination findings are described. One of the dogs underwent further clinical diagnostics. Advanced clinical diagnostics on the fellow, presumed to be non-glaucomatous eye of a dog revealed: pectinate ligament dysplasia by gonioscopy, retinal thinning in the depigmented area and wedge shaped retinal thinning with delayed choroidal vascular perfusion by optical coherence tomography, confocal scanning laser ophthalmoscopy, fluorescein and indocyanine green angiography. Quantifiable maze testing for the same eye revealed mild nyctalopia but the full-field electroretinogram showed no generalized decrease of retinal function. Genetic testing for mutations within the *retinitis pigmentosa GTPase regulator* gene causing X-linked progressive retinal atrophy in Siberian Huskies was negative. Histopathologic evaluations on enucleated eyes in two dogs confirmed goniodysgenesis, PACG with optic nerve head cupping, and diffuse inner retinal atrophy. In addition, segmental profound retinal atrophy, loss of retinal pigment epithelium, and adhesion of the retina to Bruch’s membrane was observed and coincided with multifocal depigmented lesions noted on fundic examination.

**Conclusions:**

To our knowledge, this is the first case series with clinical and histopathologic data of chorioretinal lesions, most likely caused by severely impaired choroidal perfusion. Further studies are warranted to elucidate the etiology and pathophysiology, including its possible association with PACG.

## Background

Etiologies of diseases in the canine fundus include hereditary and nonhereditary developmental anomalies, inherited retinal degenerations/dystrophies, nutritional deficiencies, infections, neoplasia, toxic chorioretinopathy, vascular diseases, immune mediated retinopathy, glaucoma, and trauma [1]. Fortunately, advancement of imaging technologies and extensive studies on clinical features, pedigree analysis, and genetics of canine inherited retinal degeneration/dystrophy have resulted in improvement of clinical diagnostic abilities and the development of genetic tests and novel therapies [1–3]. However, even with extensive diagnostics, some clinical cases with fundic abnormalities remain unclear in terms of a definite etiologic diagnosis, thus appropriate treatment options and accurate determination of long term prognosis are hard to provide. A significant number of diseases involving the canine fundus are breed-related, suggesting possible genetic components in the pathogenesis [1, 4].

Ocular disease with possible chorioretinal changes to which Siberian Huskies are predisposed to include glaucoma, uveodermatologic syndrome (UDS), X-linked progressive retinal atrophy (PRA), and retinal dysplasia [5]. The genetic mutation and mode of inheritance are yet to be determined in most of these diseases with exception of X-linked PRA. However, clinical features of each disease have been described in detail [1, 5].

Here, we report clinical and pathological data of unique chorioretinal changes in two spayed female Siberian Huskies and a Siberian Husky/Australian Shephard mixed breed dog with suspected primary angle-closure glaucoma (PACG).

## Case Presentation

### Case 1

#### History

A 4-year-old, spayed female Siberian Husky was presented to the Michigan State University (MSU) Comparative Ophthalmology service seeking a second opinion on treatment of glaucoma in the left eye (OS). The owners adopted the dog as a stray approximately one year ago. Since adoption, the dog had been on a commercially available canine adult diet with addition of cooked vegetables and meat. The dog was up to date on vaccinations and heart worm preventives, and reported to be otherwise healthy.

**Fig. 1:**
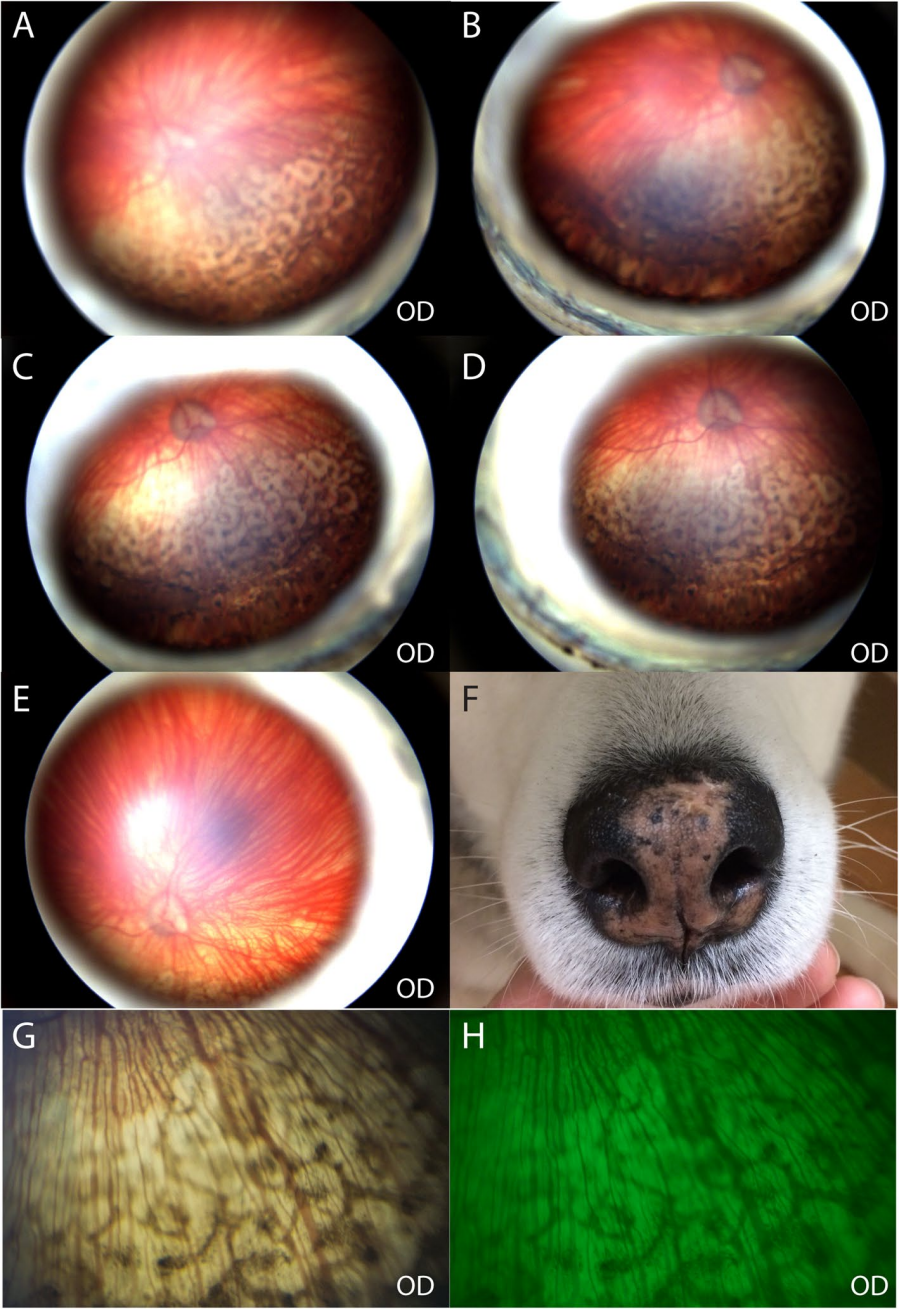
Multifocal irregular shaped depigmented lesions surrounding central pigmented lesions on the inferior fundus in a 4-year-old, spayed female Siberian Husky (case 1, OD). The dog had blue iris and an atapetal fundus OU. Both eyes were symmetrically affected with the multifocal depigmented lesions, but images were obtained from OD only due to corneal edema OS. **A** and **B** Images were taken at the first visit. The images are not sharply resolved due to the lack luster corneal surface caused by low tear production. **C**-**E** Images were taken at a recheck visit 4 months after the first visit. No grossly notable change in appearance, size and extent of the lesions from the first visit was observed. **F** The nasal planum had hypopigmentation, which went unchanged throughout the 2-year follow-up period. **G**, **H** Close-up images of the fundic lesions with (**H**) and without (**G**) a red-free filter show central pigment deposits are located over the choroidal vasculature

Approximately 4 weeks prior to this visit, the dog was diagnosed with glaucoma OS by the primary veterinarian. At that time, the intraocular pressure (IOP) was 5 mmHg in the right eye (OD) and 46 mmHg OS. Then, she was seen by a boarded veterinary ophthalmologist and diagnosed with secondary glaucoma OS with a suspected underlying primary etiology of UDS. A menace response was positive OD but negative OS. Buphthalmos and optic disc cupping with loss of myelin were observed on fundic examination OS. Additionally, both eyes had multifocal patchy round, coalescing regions of depigmentation in the inferior fundus (Fig. 1). The rest of the ophthalmic examination was unremarkable. Medical treatment was started with topical 0.005% latanoprost (OS q12h), 2% dorzolamide HCl (OS q8h), and 0.5% timolol maleate (OS q8h) ophthalmic solutions for IOP control, and 1% prednisolone acetate ophthalmic solution (OD q24h and OS q12h) combined with oral prednisone 20 mg (approximately 1 mg/kg PO q12h for 1 week followed by a tapering schedule) for an anti-inflammatory and an immune-modulatory effect to treat suspected UDS.

#### Ophthalmic examination

On the first visit to MSU, the dog received a complete ophthalmic examination. The evaluation included neuro-ophthalmic evaluations, Schirmer tear test (Schirmer tear test strips, Schering-Plough Animal health, Kenilworth, NJ, USA), fluorescein staining (Flu-Glo fluorescein sodium ophthalmic strips, Akorn, Lake Forest, IL, USA), tonometry (Icare Tonovet, Vantaa, Finland), slit-lamp biomicroscopy (Kowa SL-17 portable slit lamp, Tokyo, Japan) and binocular indirect ophthalmoscopy (Keeler binocular indirect ophthalmoscope, Broomer, PA, USA ;Volk pan retinal 2.2D, Mentor, OH, USA). The examination showed OS to be non-visual, with negative direct and consensual (from OS to OD) pupillary light reflexes (PLR). The pupil was fixed mid-dilated. Additional anterior segment findings OS included buphthalmos, moderate episcleral congestion, moderate conjunctival hyperemia, lack luster corneal surface, moderate diffuse corneal edema, 1+ aqueous flare/cells, and incipient anterior cortical cataracts. Menace response was positive OD in a room-light setting, but negative in a dim-light setting. There was no abnormality noted in the anterior segment except lack luster corneal surface OD. Fundic examination showed an atapetal fundus OU, multifocal polygonal shaped depigmented lesions, the centers of which showed pigment deposits on the inferior fundus OU and optic disc cupping OS. The inferior fundus demonstrated choroidal vascular attenuation OU. The fundic changes OD were serially photo-documented with a high-resolution ocular imaging system (RetCam, Clarity Medical Systems, Pleasanton, CA, USA; Fig. 1A-E) but lesions OS, although visible with the indirect ophthalmoscope, were not photo-documented due to the limited image quality from the corneal edema. The multifocal areas of hypopigmentation in the nasal planum that the patient had were also photo-documented (Fig. 1F). Close-up images with and without a red-free filter [modified full spectrum digital single lens reflex (dSLR) camera (Canon 7D, Canon, Tokyo, Japan), dSLR camera adaptor, camera lens (Canon EF-S 60 mm f/2.8 macro lens, Canon, Tokyo, Japan) and indirect lens 40D (Volk), red-free filter (MF525/39 nm, Thorlabs, Newton, NJ)] showed central pigment deposits located over the choroidal vasculatures (Fig. 1G and H). Based on funduscopic examination and high-resolution imaging, depigmented lesions were thought to be from a lack of pigment in the RPE layer with central RPE pigment hypertrophy.

**Fig. 2:**
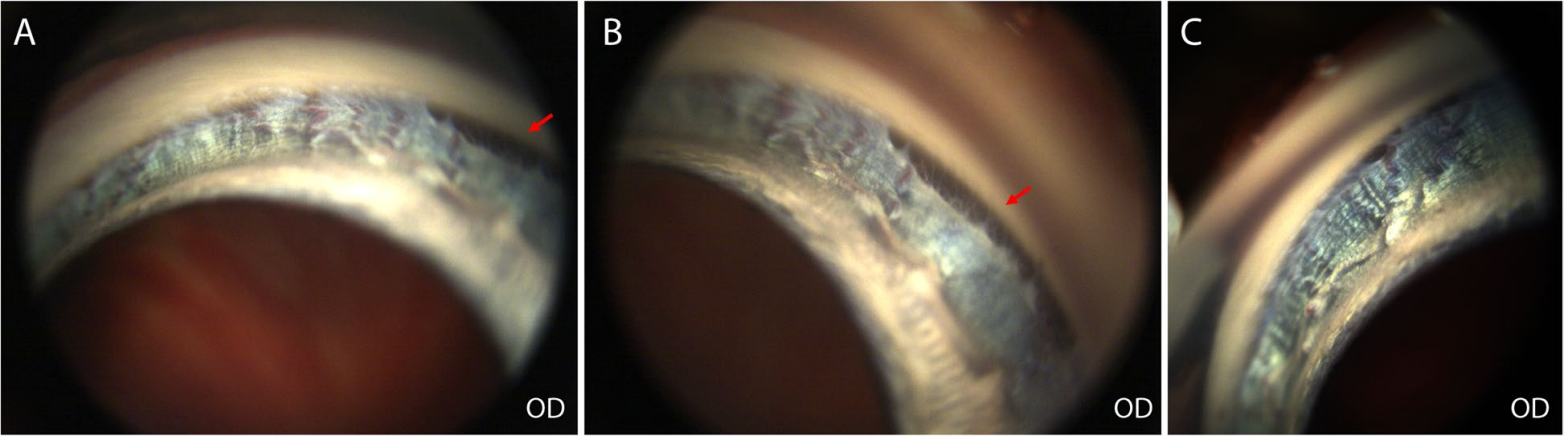
Narrow iridocorneal angle with moderate pectinate ligament dysplasia was observed in a 4-year-old, spayed female Siberian Husky (case 1, OD). Segmental areas of open angle with normal pectinate ligament (red arrows) were observed in approximately less than 30% of the angle

IOP measured with a rebound tonometer was 17 mmHg OD and 53 mmHg OS. Schirmer tear test values were 4 mm/min OD and 12 mm/min OS. Gonioscopy was performed OD and recorded with a high-resolution ocular-imaging system (RetCam). The iridocorneal angle OD was narrow with moderate pectinate ligament dysplasia characterized by broad-based pectinate ligament strands and solid sheets affecting approximately 70% of the angle **(**Fig. 2**).** Due to corneal edema, the iridocorneal angle (ICA) OS was not able to be examined.

Chromatic PLR (Melan-100, BioMed Vision Technologies, Ames, IA, USA) OD showed a slow incomplete response to the red light and brisk complete response to the blue light which was suggestive of decreased photoreceptor function [6, 7]. OS was not able to be tested due to corneal edema.

#### Quantifiable maze testing

**Fig. 3:**
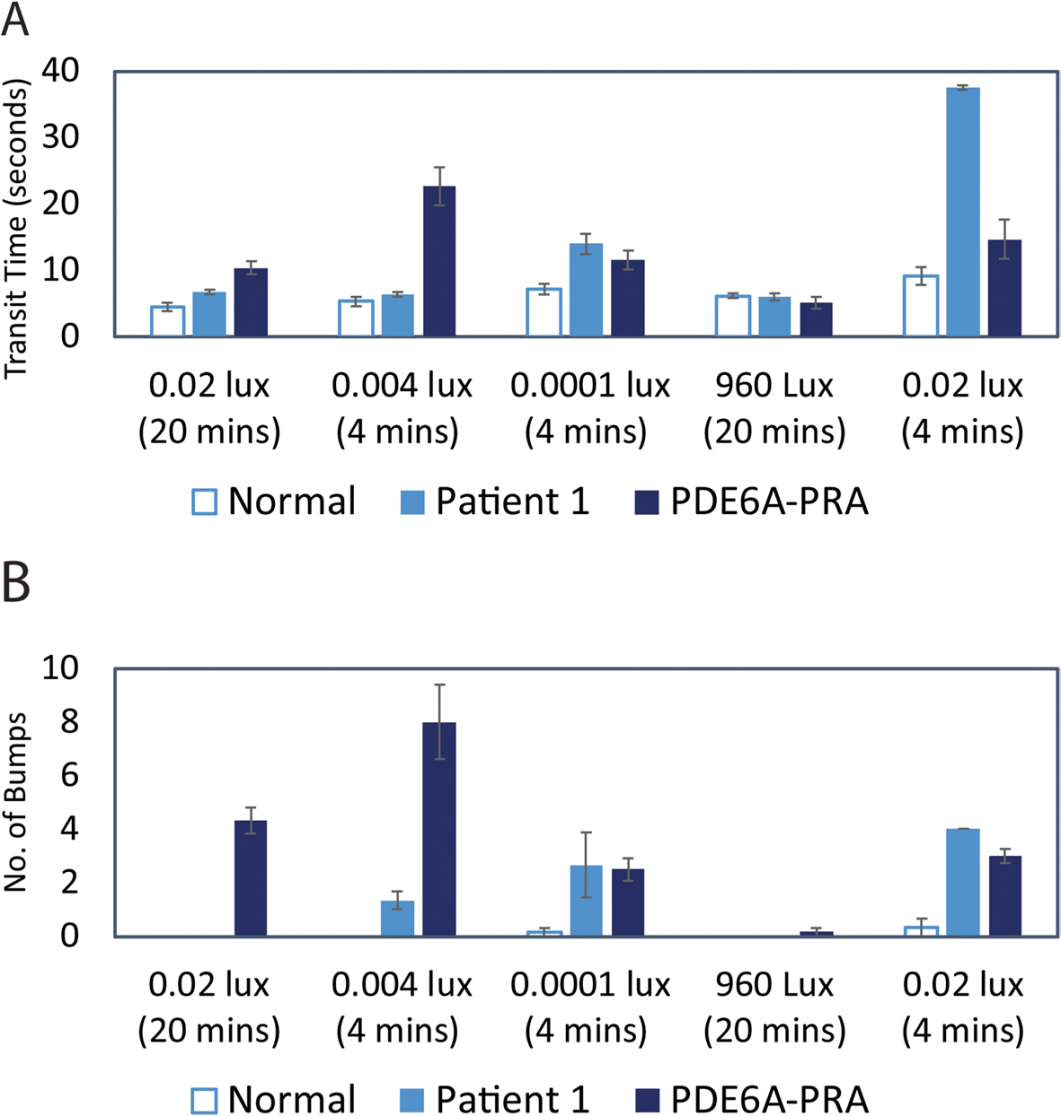
Transit time (**A**) and number of bumps (**B**) in a 4-year-old, spayed female Siberian Husky (case1, OD) are higher than normal dogs but lower than the dogs with no rod function with *PDE6A* mutation in most dim light setting. When the test was repeated with the light setting of 0.02 Lux after a short dark adaptation time (4 mins), the dog in case 1 showed markedly increase in transit time and number of bumps. Time of dark or light adaptation for each light setting is indicated in parentheses in minutes (mins)

Scotopic and photopic vision OD was tested with a custom-made obstacle-avoidance course with controlled luminance settings. Details of the course setting and the testing method have been described previously [8]. Briefly, the dog was released at the entrance and allowed to walk through an obstacle-avoidance course. Transit time and number of bumps were recorded from the video recordings and then averaged from three runs with each approximate luminance setting at the order of: 0.02, 0.004, 0.0001, 960 and 0.02 Lux. The obstacle panel position was randomly changed for each run. In most dim light settings (0.02, 0.004, and 0.0001 Lux) with initial 20 min dark adaptation and additional 4 min adaptation for each change in setting, the dog took a longer transit time and had more bumps than average age-matched normal dogs but a shorter transit time and fewer bumps than purpose-bred research dogs with advanced progressive retinal atrophy (rcd3) with no rod function caused by *PDE6A* mutation (Fig. 3 )[2]. When the test was repeated with 0.02 Lux setting after allowing a shorter dark adaptation time (4 mins) following a run with a bright luminance setting (960 Lux), the patient showed a notably poor performance compared to dogs with dogs with *PDE6A* mutation as well as her own records with the same light setting after 20-min dark adaptation (Fig. 3). The number of bumps and transit time of the patient was also markedly higher with 0.02 Lux after 4-min dark adaptation compared to her own records with the same light setting after 20-min dark adaptation. The results of maze testing suggested that this dog had mild to moderate nyctalopia following shorter dark adaptation time.

#### Electroretinography and genetic testing

**Fig. 4:**
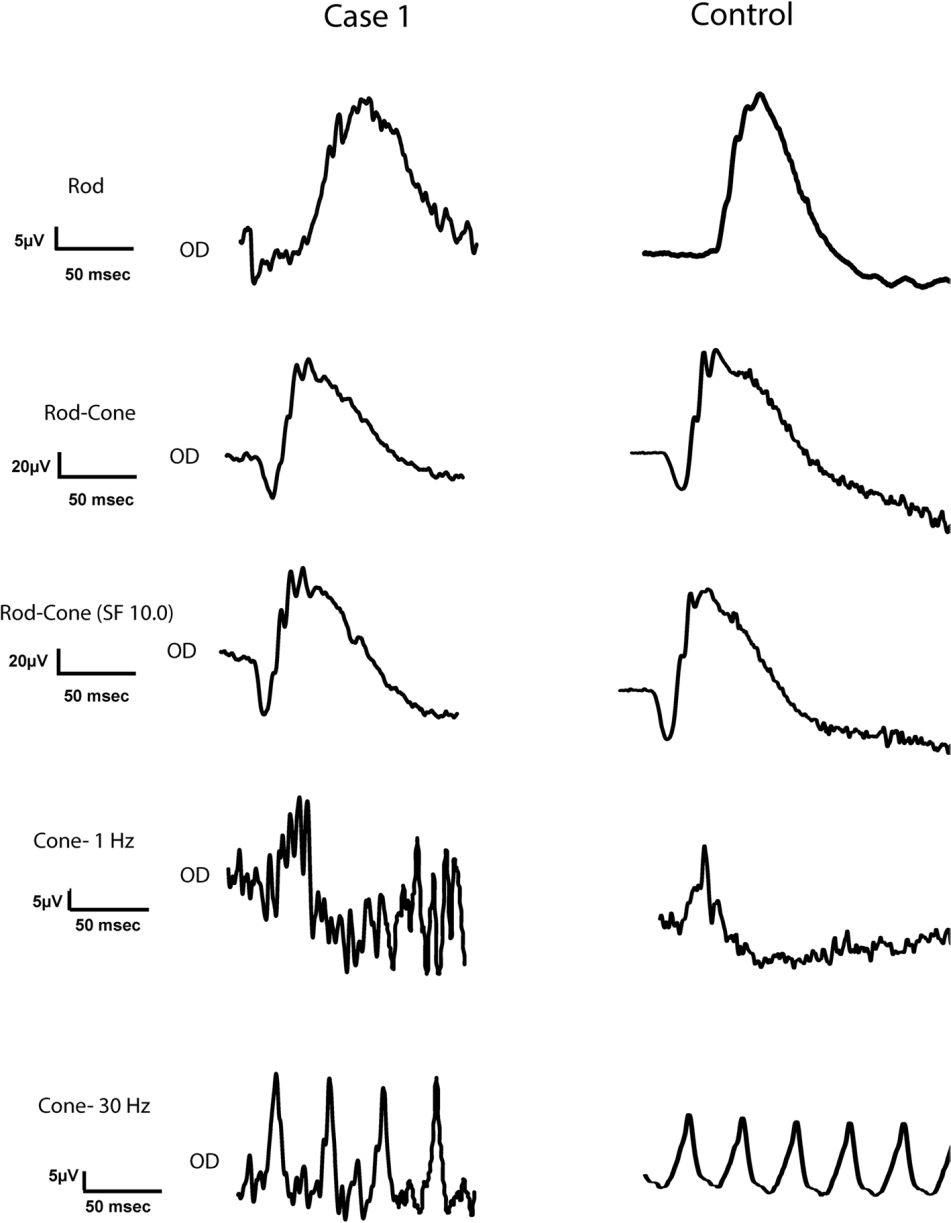
Normal electroretinography waveforms of a 4-year-old, spayed female, Siberian Husky (case 1, OD) with bilateral multifocal chorioretinal lesions and a 1-year-old normal, intact male, beagle with no ocular disease

Standard full-field scotopic and photopic electroretinography (ERG) was recorded OD (RETIport ERG system, Ronald Consult, Brandenburg a.d. Havel, Germany with a mini Ganzfeld stimulator, MINIGanzfeld E8, Ronald Consult, Brandenburg a.d. Havel, Germany; a Jet Electrode, Fabrinal, Eye Care, LA Chaux-De-Fonds, Switzerland and subdermal needle electrodes, Genuine Grass, Natus neurology, Galway, Ireland) under sedation with dexmedetomidine 2 μg/kg IV (Dexdomitor, Orion Corporation, Espoo, Finland). Rod- (0.01 cd.s/m^2^) and mixed rod-cone-mediated (3 and 10 cd.s/m^2^) responses were recorded after 20 mins of dark adaptation. After 5 mins of light adaptation, a single flash (3 cd.s/ m^2^) and 30 Hz flicker (3 cd.s/ m^2^) cone-mediated responses were recorded. The ERG results were unremarkable, suggesting no diffuse pathology of the retina (Fig. 4, Table 1). For comparison, Fig. 4 and Table 1 present the ERG curves and results from a normal purposed-bred dog recorded with the same system.

**Table 1:** Electroretinography results for a 4-year-old, spayed female Siberian Husky (Case 1) with bilateral multifocal chorioretinal lesions and a 1-year-old normal, male beagle with no ocular disease

	Light Intensity (cd.s/m^2^)	Wave	Amplitude (μV)		Implicit Time (ms)	
			Patient	Normal	Patient	Normal
Scotopic	0.01	b	35.5	24.7	65.5	48.5
	3	a	29.4	27.8	13.2	14.9
		b	104	99.5	35.8	36.0
	10	a	41.8	36.5	11.2	14.1
		b	110	112	35.8	40.7
Photopic	3 (single flash)	a	7.36	5.08	11.2	13.3
		b	27.5	28.0	29.1	31.3
	3 (30 Hz flicker)	N1-P1	42.6	19.8		

A blood sample was submitted for commercially available DNA testing for the mutation in *retinitis pigmentosa GTPase regulator* (*RPGR*) responsible for X-linked PRA in Siberian Huskies (Optigen, Ithaca, New York, USA). The test result revealed the dog was homozygous for the wild-type allele and therefore not affected by XLPRA.

#### Optical Coherence Tomography (OCT)

**Fig. 5:**
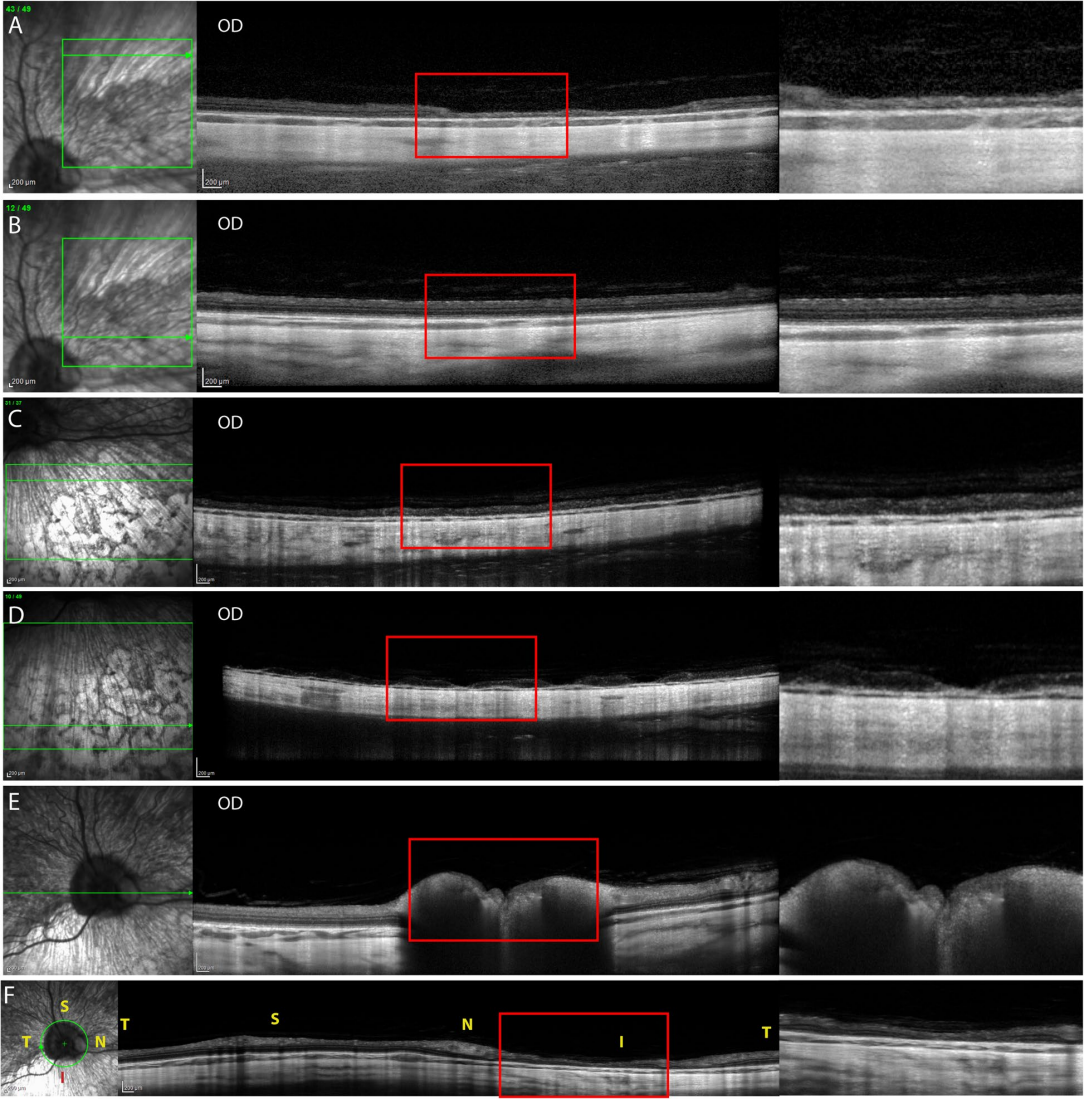
Optical coherence tomography (OCT) images of **A** peripheral superonasal, **B** central superonasal, **C** inferonasal, **D** inferior, **E** horizontal including the optic nerve head, and **F** peripapillary imaging planes of a 4-year-old, spayed female, Siberian Husky (case 1, OD) showing multifocal partial-to-complete loss of retinal thickness and structure. Note the choroidal thinning and vasculature attenuation (decreased in lumens) in the inferior sections (**C** and **D**) compared to the superior sections (**A** and **B**). Green horizontal arrows in each infrared confocal scanning laser ophthalmoscopy (cSLO) images indicate the location of the OCT imaging plane. Letters of ‘S’, ‘T’, ‘I’, and ‘N’ in the image indicate superior, temporal, temporal, and nasal quadrant of the fundus. Magnified images of the area within the red rectangle in each image are shown at the right-hand side

**Fig. 6:**
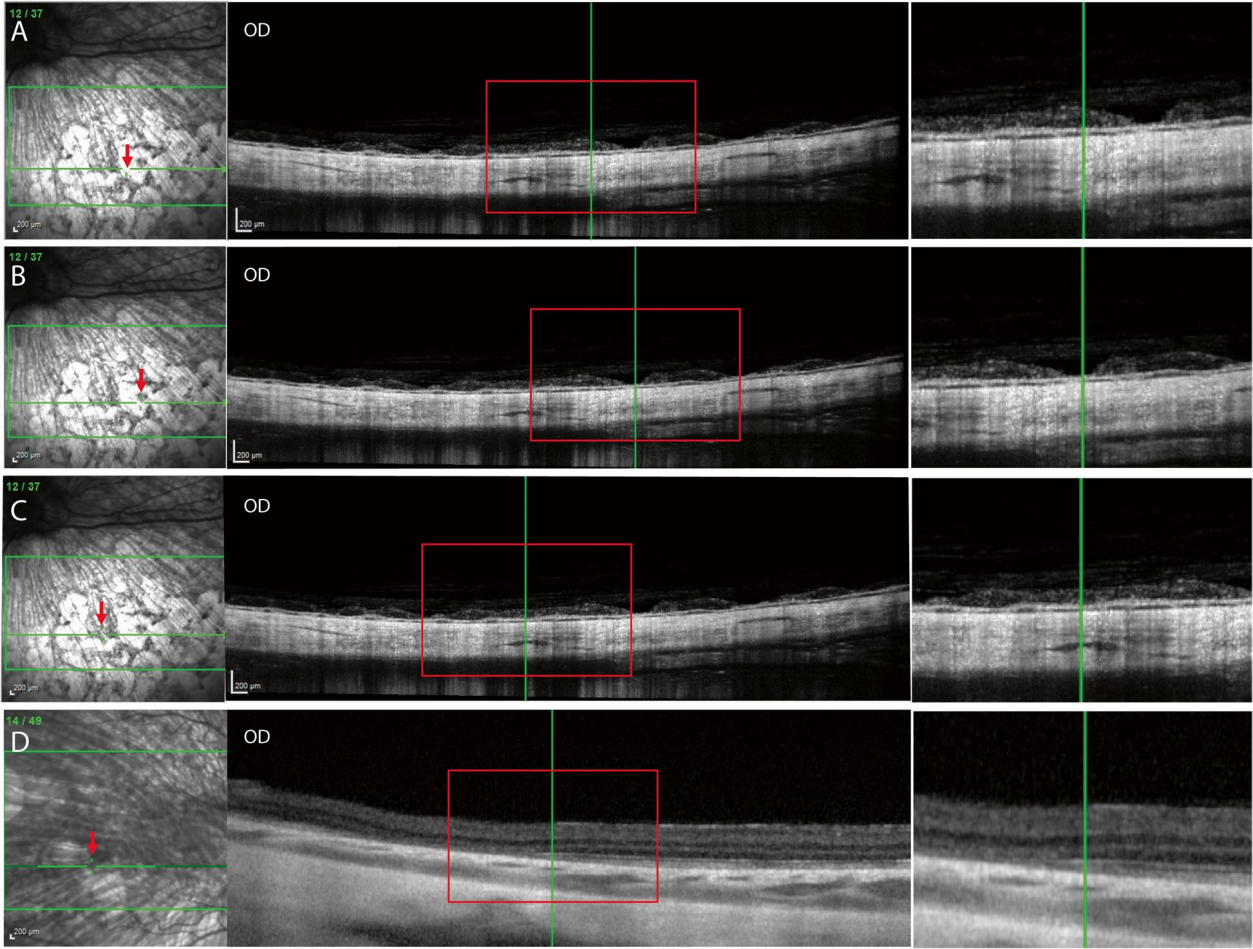
Optical coherence tomography (OCT) images of **A** depigmented area, **B** central pigment deposits, **C** surrounding area, and **D** an area of partial loss of retinal layers of a 4-year-old, spayed female, Siberian Husky (case 1, OD). Note the choroidal thinning and vasculature attenuation (decreased in lumen sizes) in the inferior sections (**A**-**C**). Green horizontal arrows in each infrared confocal scanning laser ophthalmoscopy (cSLO) image indicate the location of the OCT imaging plane. The red arrow in each cSLO image indicates the location of the vertical green line in the corresponding OCT image. An area of partial loss of retinal layers (**D**) is observed in the OCT image of the temporal fundus that appears bright in the cSLO image. Magnified images of the area within the red rectangle in each image are shown at the right-hand side

**Fig. 7:**
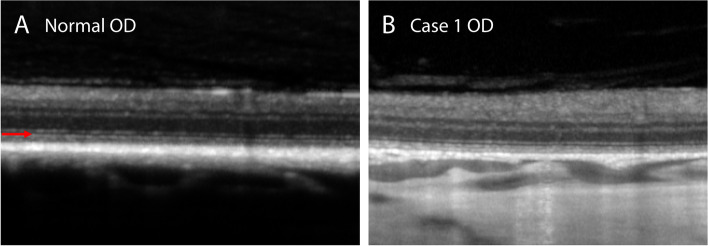
Optical coherence tomography (OCT) images show relatively intact ellipsoid zone (red arrow) in a 4-year-old, spayed female, Siberian Husky (case 1, OD) with multifocal depigmented chorioretinal lesions. **A** For comparison, an OCT image of an intact ellipsoid zone in the superotemporal fundus in a 5-year-old, intact female, mixed-breed dog with no ocular disease (normal OD). **B** The image was obtained from the superotemporal fundus close to the optic nerve where the retina was relatively well-preserved without depigmented chorioretinal lesions (case 1 OD)

High resolution SD-OCT images (Spectralis HRA/OCT, ver. 5.1.3.0, Heidelberg Engineering, Germany) of the ocular fundus were acquired from OD under general anesthesia. The patient was pre-medicated for anesthesia with acepromazine maleate (0.03 mg/kg IM, PromAce Injectable, Boehringer Ingelheim, St. Joseph, MO, USA) and butorphanol tartrate (0.4 mg/kg IM, Torbugesic, Zoetis, Kalamazoo, MI, USA). Anesthesia was induced with propofol (3 mg/kg IV, PropoFlo, Zoetis, Kalamazoo, MI, USA) and inhalation gas anesthesia was maintained with isoflurane (Isothesia, VetUS, Dublin, OH, USA) in 100% oxygen. Horizontal volume and line scans were obtained from the superotemporal (ST), superonasal (SN), inferior (I), inferotemporal (IT), inferonasal (IN), peripapillary (PP) and optic nerve head (ONH) areas. OCT images revealed areas of multifocal depigmentation in the inferior fundus associated with partial to full thickness retinal atrophy (Fig. 5C and D): the central pigmented areas had full thickness and the depigmented surrounding areas had partial thickness loss and disruption of the retinal layers (Fig. 6A-C). Partial loss of retinal thickness was observed superior to the depigmented lesions in the inferior retina where no gross abnormality was observed on indirect ophthalmoscopy (Fig. 5C). The superior retina near the optic nerve appeared to have maintained a relatively normal structure (Fig. 5B). Within this relatively well-preserved area, the ellipsoid zone (photoreceptor inner segment/outer segment junction) appeared to be intact (Fig. 7). Additional findings included segmental partial thickness loss of the retina within the superonasal (Fig. 5A) and the superotemporal quadrants (Fig. 6D). These areas did not show any gross change on indirect ophthalmoscopy. The optic nerve head appeared normal (Fig. 5E). Peripapillary scan revealed a relatively thin retinal nerve fiber layer in the inferior retina (Fig. 5F). Additionally, the choroid was also relatively thinner in the inferior region (Figs. 5C and D and 6A-C) compared to the superior region (Fig. 5A, B). The lumens of the choroidal vessels were decreased in size which was seen as lack/decrease of hyporeflective regions within the choroid which is usually prominent in the color dilute eye. This indicated some choroidal vasculature attenuation.

#### Infrared imaging and fluorescein and indocyanine green angiography of the fundus

**Fig. 8:**
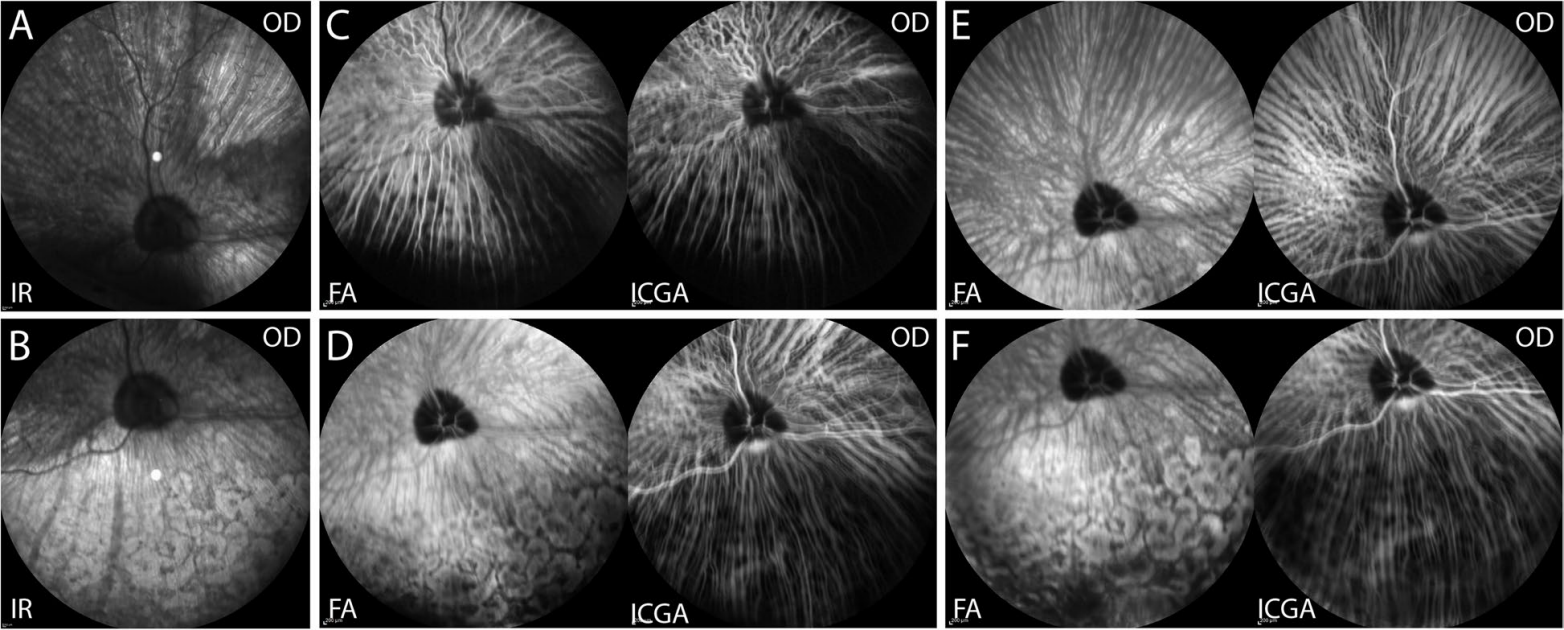
**A** An Infrared (IR) image of the superior fundus; **B** an IR image of the inferior fundus; **C** fluorescein angiography (FA, left) and indocyanine green angiography (ICGA, right) images with arterial phase with choroidal filling; **D** FA (left) and ICGA (right) images with arteriovenous phase; **E** and **F** FA (left) image with late venous phase for ICGA and staining phase for FA of a 4-year-old, spayed female, Siberian Husky (case 1, OD). Wedge-shaped lesions were observed in the superonasal fundus with infrared (IR) imaging (**A**) and inferonasal fundus with fluorescein (left in **C** and **D**) and indocyanine green angiography (ICGA, right in **C** and **D**). Multifocal hypopigmented areas in the inferior fundus exhibited hyperfluorescence in fluorescein angiography (FA, left in F) but hypofluorescence in ICGA in the late phase (right in **F**)

Following the OCT, additional infrared confocal scanning laser ophthalmoscopy (cSLO) images of fundus OD were taken (Spectralis). The cSLO images showed a wedge-shaped lighter/hyperreflective lesion surrounded by a dark delineation in the superonasal fundus (Fig. 8A). This area corresponded to the area of retinal atrophy in the superonasal fundus observed with OCT (Fig. 5A). The depigmented areas in the inferior fundus appeared brighter in the cSLO imaging, which suggested more reflection from the sclera due to a thinner overlying retina and choroid (Fig. 8B). Following the cSLO imaging, a combination of fluorescein (AK-Fluor 10%, Akorn, Lake Forest, Illinois, USA; 20 mg/kg) and indocyanine green (ICG, IC-Green, Akorn, Lake Forest, Illinois, USA; 1 mg/kg) were injected IV and approximately 3 consecutive 1-min-long angiogram videos were recorded. Then, still images were taken in different areas. A delayed choroidal filling defect was observed in a wedge-shaped area of inferonasal fundus in the pre-arterial, arterial (Fig. 8C), and arteriovenous (Fig. 8D) phases. In the late phase of fluorescein angiography (FA), hyperfluorescence was observed in the depigmented lesions, likely due to a window defect caused by an absence of RPE pigment and partial loss of the retina; while those areas appeared dark in indocyanine green angiography (ICGA), suggestive of reduced perfusion/vasculature (Fig 8E and F).

#### Differential diagnoses

Clinical findings were consistent with chronic glaucoma OS and pectinate ligament dysplasia OD, suggested PACG. The dog also had low tear production OU. However, the diagnosis of the bilateral fundic lesions and their correlation with glaucoma were unclear. Our differential diagnoses for the fundic lesions included immune-mediated chorioretinitis (i.e., UDS), developmental anomaly (i.e., hypoplasia or coloboma), inherited (i.e., PRA), secondary lesions to glaucoma/ischemia, or nutritional deficiency (i.e., previous vitamin E deficiency) based on the ophthalmoscopic appearance of the lesions and the history of being found as a stray dog.

#### Treatments

Based on the gonioscopy results and with the aim of delaying glaucoma onset OD, a prophylactic IOP-lowering and anti-inflammatory treatment was started which included 2% dorzolamide HCl-0.5% timolol maleate ophthalmic solution (Hi-Tech Pharmacal, Amityville, NY, USA—OD q12h) and diclofenac 0.1% ophthalmic solution (Akorn, Lake Forest, IL, USA—OD q24h )[9]. Treatment for low tear production consisted of cyclosporine 1% ophthalmic solution (compounded at MSU Veterinary Medical Center Pharmacy, East Lansing, MI, USA – OD q12h). Enucleation OS was performed under routine general anesthesia for pain control in the blind eye and the globe was submitted for histopathologic evaluation. After the enucleation procedure, 2 small biopsy samples were taken from the area of hypopigmentation in the nasal planum (Fig. 1F), for a potential diagnosis of UDS.

#### Histopathologic evaluation

**Fig. 9:**
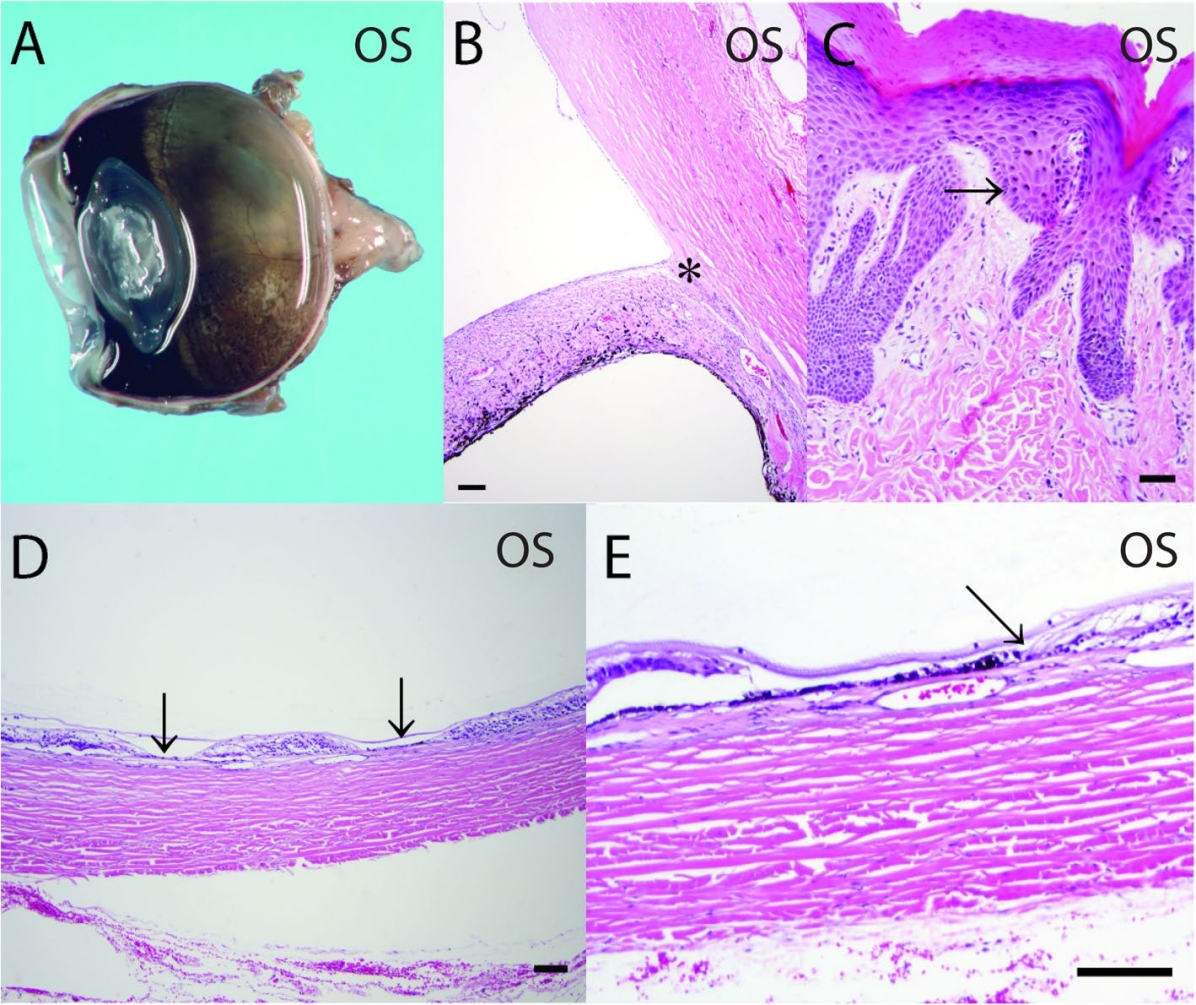
**A** Gross image of the enucleated globe showing multifocal reticular shaped depigmented lesions in a 4-year-old, spayed female, Siberian Husky (case 1, OS), the centers of which showed pigment deposits involving the inferior fundus. **B** A broad, non-perforate, sheet-like band of uveal stroma (asterisk) bridges from the base of the iris to the terminal arborization of Descemet's membrane and extends over the collapsed ciliary cleft. **C** Histopathologic examination of biopsied nose pad showed variable melanin pigment presence within the basal keratinocyte layers of the epidermis (melanin indicated by arrow). **D** Histopathologic evaluation OS revealed severe diffuse inner to segmental profound (arrows) retinal atrophy which was consistent with the OCT findings in the fellow eye (OD), which never had a documented IOP spike. **E** There is multifocal loss of the retinal pigment epithelium and adherence of the neural retina to Bruch's membrane (arrow). The choroid is thin and atrophic. Hematoxylin and eosin (H&E) stain. Scale bars for **B**, **D**, and **E** = 50 μm and for C = 20 μm

Gross- and histopathologic evaluation of OS revealed suspected goniodysgenesis with collapsed ciliary cleft, mild pigment dispersion, preiridal cellular to fibrous membrane, choroidal thinning and atrophy, rare axonal degeneration in the optic nerve, diffuse inner retinal atrophy, and segmental profound retinal atrophy (Fig. 9A, B and D, E). In the non-tapetal fundus with full thickness retinal atrophy, multifocal infiltrates of pigmented cells into the neural retina were observed. Multifocal loss of the RPE and adherence of the neural retina to Bruch's membrane were also noted. Nasal planum biopsy showed variable melanin pigment presence within the basal keratinocyte layers of the epidermis suggesting leukoderma or vitiligo (Fig. 9C). There were no indications of an underlying infectious, neoplastic, or autoimmune etiology in the globe or nasal planum samples (Fig. 9). These findings were consistent with chronic PACG without any evidence of uveodematologic syndrome. The etiology for the multifocal retinal atrophy was not clear.

#### Follow-up

Since initial presentation to MSU, recheck examinations have been performed every 1-3 months. The IOPs OD have been maintained within normal range (8 – 14 mmHg), and no gross progressive changes have been observed on ophthalmic examinations (Fig. 1C, D, and E). On the most recent examinations—performed 2-year following initial presentation— menace response was positive OD in a room-light setting, but negative in a dim-light setting, which was subjectively unchanged from the initial visit. At follow-up visits, the dog was not subjected to quantifiable maze testing. There have been no clinical sign of active inflammation. STT OD was measured at 13 mm/min and signs of xerosis on the cornea have markedly improved.

### Case 2

**Fig. 10:**
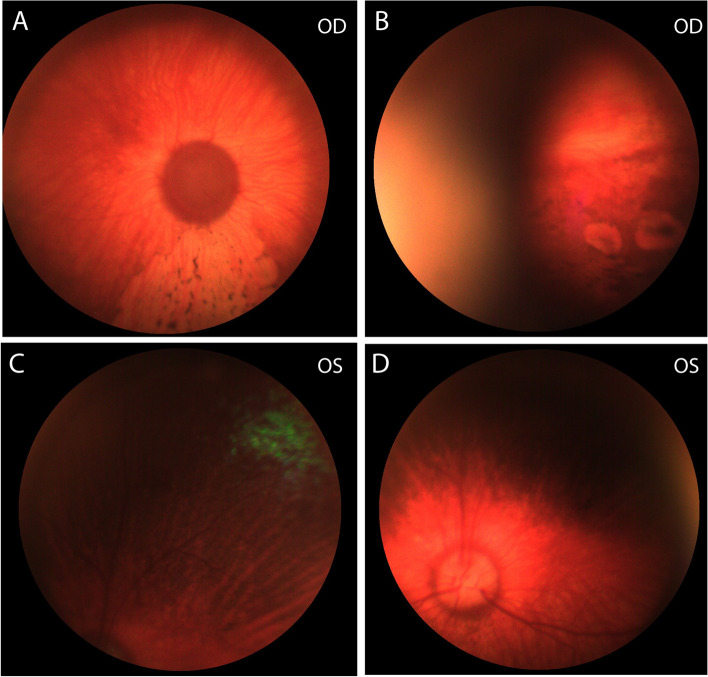
**A** Wedge-shaped area without retinal vessels and decreased central RPE pigmentation with focal areas of increased pigmentation surrounding the periphery of noted lesion within the superotemporal fundus of a 3-year old, spayed female, Siberian Husky with glaucoma (case 2, OD). Optic disc cupping and demyelination can be observed. **B** Two small, circular lesions with lack of pigmentation in the inferotemporal fundus OD but underlying choroidal vessels were observed. **C** Superotemporal fundus (OS) showing a small area of tapetal fundus. **D** No significant fundic lesion was observed OS except for slight pallor of the optic nerve head

A 3-year-old, spayed female Siberian Husky was diagnosed with primary glaucoma OU and medically managed by the Ophthalmology Services at Colorado State University (CSU) Veterinary Teaching Hospital and VRCC Veterinary Specialty and Emergency Hospital. At the time of first examination at CSU, the OD was blind but OS was visual. The IOP was 34 mmHg OD and 13 mmHg OS measured with a Tono-Pen Avia® Vet (Reichert Inc., Depew, NY 14843). Gonioscopy OS (Koeppe Goniolens, Ocular, Bellevue, WA 98004) revealed no visible pectinate ligament with closed iridocorneal angle. Indirect ophthalmoscopy and fundic imaging (Optibrand’s Clearview Fundic Camera, Fort Collins, CO) revealed an atapetal fundus OD (Fig. 10A and B) and a small tapetum OS (Fig. 10C). In the inferior fundus OD, there was a wedge-shaped area without retinal vessels and decreased central RPE pigment with focal areas of increased pigment (Fig. 10A). Two small circular lesions with lack of pigmentation were observed in the inferotemporal fundus OD, but the underlying choroidal vessels were visible (Fig. 10B). The optic disc appeared dark and cupped OD (Fig. 10A) and normal in size but pale OS (Fig. 10D). No chorioretinal lesions were noted OS throughout the 8-month of follow-up.

**Fig. 11:**
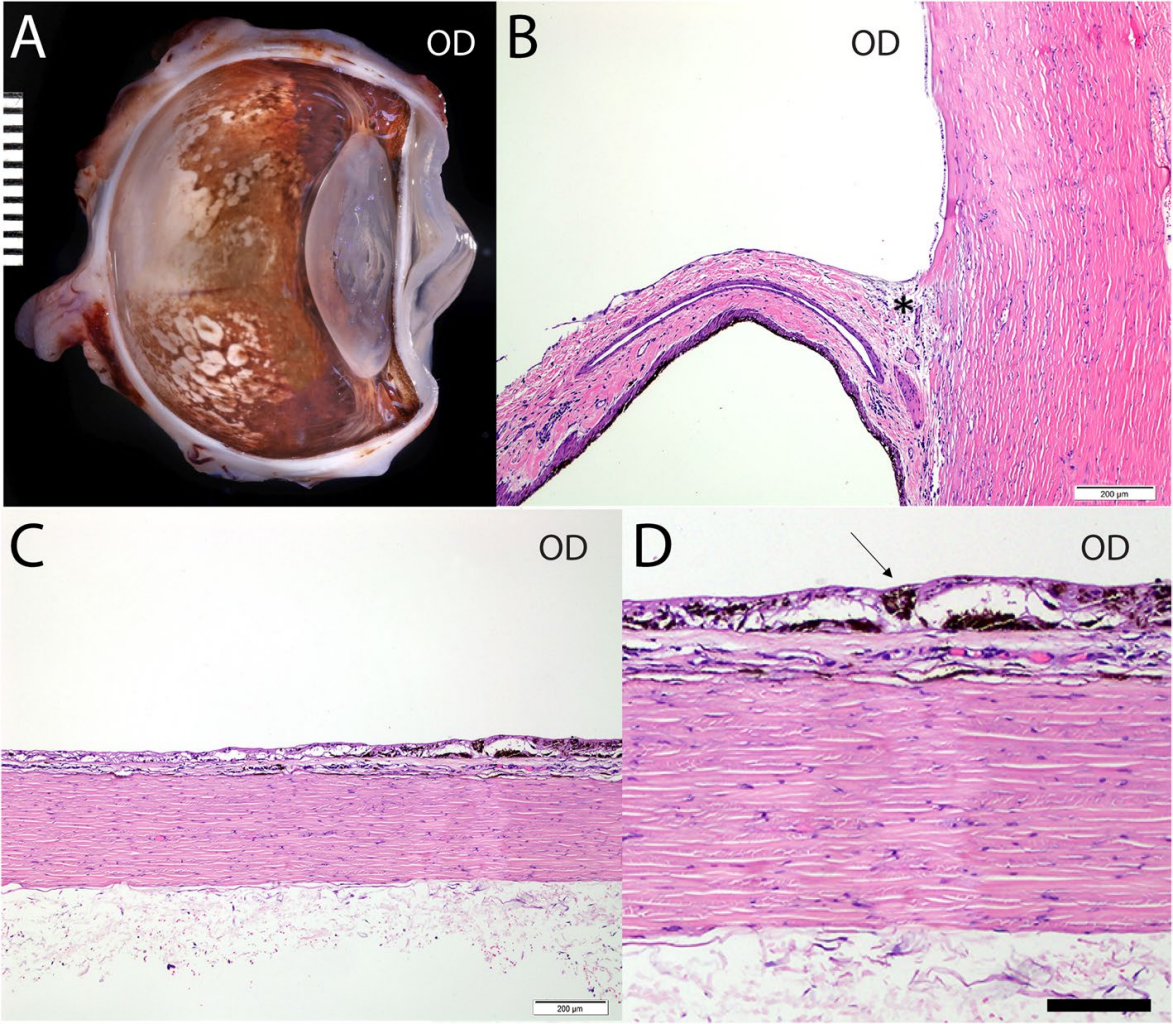
**A** Gross image of enucleated globe of a 3-year old, spayed female, Siberian Husky (case 2, OD) shows multifocal reticular shaped depigmented lesions, the centers of which showed pigment deposits on the peripheral superior and most of inferior fundus. **B** A broad, non-perforate, sheet-like band of uveal stroma (asterisk) bridges from the base of the iris to the terminal arborization of Descemet's membrane and extends over the collapsed ciliary cleft. **C** and **D** Histopathologic evaluation OD revealed severe diffuse inner to segmental profound retinal atrophy. There is multifocal loss of the retinal pigment epithelium and adherence of the neural retina to Bruch's membrane. The choroid is thin and atrophic. Hematoxylin and eosin (H&E) stain. Scale bars for B and C = 200 μm and for D =100 μm

Medical treatments included 2% dorzolamide HCl (Akorn, Lake Forest, Illinois, USA, OU q8h), 0.5% timolol maleate (Akorn, Lake Forest, Illinois, USA, OU q12h), and 0.005% latanoprost ophthalmic solutions (Bausch and Lomb, Bridgewater, NJ, USA – OU q12h). OD was enucleated approximately 7 months after the initial diagnosis due to uncontrolled IOP. Gross and histopathologic evaluation revealed goniodysgenesis, buphthalmos, iridocorneal angle descemetization, pre- and post-iridal fibrovascular membranes, mild lymphoplasmacytic anterior uveitis, trabecular meshwork pigment dispersion, and broad posterior synechiae (Fig. 11). While follow up fundus images were not obtained, comparison of images from the initial examination with the gross eye image after enucleation suggested progression of the chorioretinal lesions (Fig. 11A). Similar to case 1, diffuse full thickness atrophy of the choroid and retina, RPE degeneration, hypertrophy and hyperplasia with sub- and intra-retinal pigment dispersion were also observed (Fig. 11B and C).

### Case 3

**Fig. 12:**
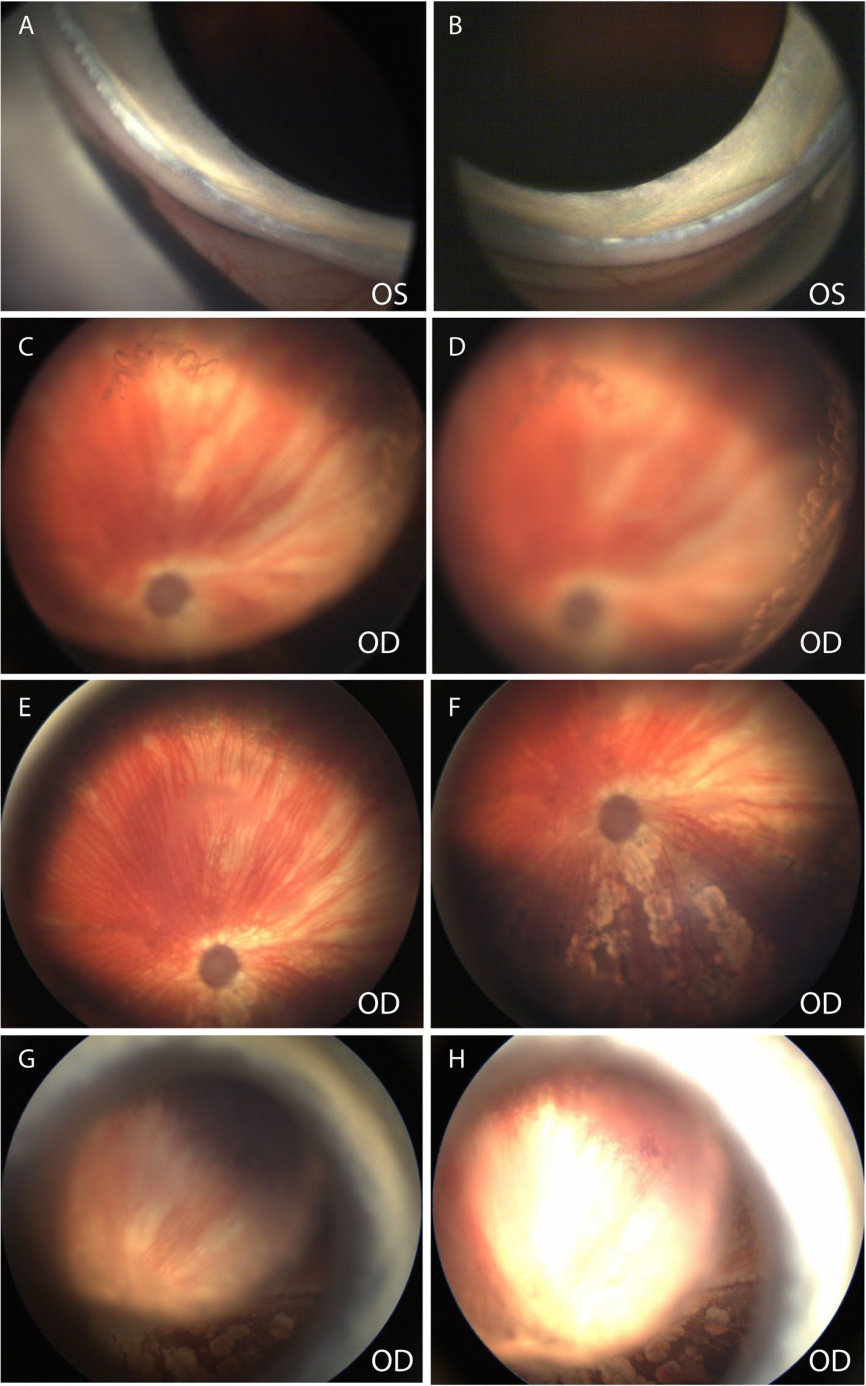
**A** and **B** Narrow iridocorneal angle with moderate pectinate ligament dysplasia was observed in an 11-month-old, spayed female Siberian Husky/Australian Shepherd mix (case 3, OS). **C** Posterior cortical and **D** equatorial cataracts with vacuoles are observed (OD). **E** Wedge shaped areas with choroidal vessel attenuation in the superior fundus and **F** with multifocal irregular shaped depigmented lesions surrounding central pigmented lesions on the inferior fundus OD. **A**-**F** Images were obtained at the initial visit. **G** and **H** Funduscopic images OD taken 11 months after the initial visit revealed increased area of depigmented lesions. The image is not sharply resolved due to the progressed cataract

An 11-month-old, spayed female Siberian Husky-Australian Shepherd mixed breed dog was diagnosed with primary glaucoma OD and pectinate ligament dysplasia OU (Fig. 12A and B) and medically managed by the MSU Comparative Ophthalmology service. At the time of first examination OD was blind but OS was visual. The IOP was 18 mmHg OD and 7 mmHg OS measured with a rebound tonometer. Gonioscopy was performed OS and recorded with a high-resolution ocular-imaging system (RetCam, Fig. 12A and B) and revealed severe pectinate ligament dysplasia. OD had buphthalmos, posterior subcapsular cataract with equatorial vacuoles (Fig. 12C and D). Aphakic cresent was also observed OU. Indirect ophthalmoscopy and fundic imaging revealed an atapetal fundus OU (Fig. 12E and F), a wedge-shaped area OD with multifocal polygonal shaped depigmented lesions, the centers of which showed pigment deposits and choroidal vessel attenuation, 2 wedge-shaped areas with choroidal vessel attenuations in the superior and superonasal fundus, and optic disc cupping OD (Fig. 12E and F). The fundus OS was normal over the entire follow-up period. Medical treatments included 2% dorzolamide HCl-0.5% timolol maleate (Hi-Tech Pharmacal, Amityville, NY, USA—OU q12h) and latanoprost (Bausch and Lomb, Bridgewater, NJ, USA – OD q12h). Over the follow-up period of 11 months, despite no recorded IOP spikes, the chorioretinal lesions progressed with an increased number of wedge shaped areas with choroidal vessel attenuation in the superior and inferior fundus and pigment changes in the inferior fundus (Fig. 12G and H).

## Discussion and conclusions

We report three comparable cases of glaucoma with unique multifocal hypopigmented chorioretinal lesions in two Siberian Huskies and a Siberian Husky/Australian Shepherd mixed breed dog with extensive clinical diagnostic data in one of these dogs, including color and infrared cSLO fundic images, FA, ICGA, OCT, quantitative maze test, ERG, genetic test, and histopathology. All dogs were young adult spayed females between 11 months and 4 years of age. This report provides detailed clinical and pathological documentations of multifocal hypopigmented retinopathy associated with PAG in two purebred and a mixed Siberian Huskies.

### Paving-stone retinal degeneration in humans

Comparable retinal lesions as noted in the current case series have been described as paving-stone or cobblestone retinal degeneration in humans [10, 11]. The funduscopic and OCT images as well as histologic description reported in humans are consistent with observations in our canine patients [10–14], with multiple, small, well-demarcated flat lesions. Irregular black pigmentation is frequently present along the margins of the lesions. OCT and histology in humans have demonstrated marked thinning of the neural retina with RPE loss and with RPE hyperplasia surrounding these regions [10–13]. It has been suggested that these changes are associated with choroidal vascular insufficiencies and are consistent with OCT and angiographic findings in our case 1. In humans, similar chorioretinal lesions have also been reported in patients with pathologic myopia, inherited retinal dysplasia, such as gyrate atrophy, and choroidal neoplasms, including malignant melanoma and metastatic carcinoma [12, 14–16]. However, approximately 15% of autopsied human eyes with or without other ocular pathology were also reported to have paving-stone degeneration, and 38% of the cases were bilateral [12]. Therefore, direct relationship of paving-stone degeneration to any specific ocular diseases remains unknown.

### Differential etiological diagnoses

Siberian Huskies are predisposed to develop primary glaucoma with pectinate ligament dysplasia; this condition has been reported more frequently in young and middle-age females [17, 18]. However, the mode of inheritance or genetic mutation associated with this disease has not been determined [17]. While cases presented in the present report were all young spayed females with blue irides that perfectly fit previous descriptions of this breed’s predisposition for primary glaucoma, chorioretinal changes observed have not been reported to be associated with glaucoma in Siberian Huskies or in any other breed. A variety of diagnoses can be made based on the multifocal depigmented retinal lesions in this breed. Possible etiological diagnoses include developmental anomaly, immune-mediated disease, inherited PRA/dystrophy, nutritional deficiency, secondary to glaucoma/ischemia, or any combination of these.

### Differential diagnosis: developmental anomalies

Developmental anomalies of the fundus reported in dogs include Collie eye anomaly, with choroidal hypoplasia, optic disc coloboma, and/or retinal detachment, merle ocular dysgenesis, retinal dysplasia, and canine multifocal retinopathy [1]. Chorioretinal colobomas also have been reported in human patients, but they are usually accompanied by retinal detachment [19, 20]. Recently, a chorioretinal coloboma has been reported in a Golden Retriever with FA and OCT data [21]. Although clinical signs and diagnostic results do not resemble any of the reported developmental anomalies in dogs, abnormal development of the RPE and neuroretina is a possibility, especially given the subalbinotic features of the dogs described herein.

### Differential diagnosis: inflammation

Chronic inflammation, including immune-mediated chorioretinitis, has been described to cause multifocal depigmentation with retinal and choroidal vessel attenuation [22]. RPE pigment migration in the center of depigmented lesions suggest that those lesions are potentially post-inflammatory. Another possible differential was UDS given the clinical presentation of depigmented fundic changes combined with vitiligo on the nasal planum; Siberian Huskies are predisposed to this disease [5]. UDS was considered less likely to play a role in our cases because of no significant anterior uveitis or evidence of active inflammation in the histopathologic evaluation of the two globes and nasal planum. Immune-mediated etiology cannot, however, be completely excluded because one of the dogs was on a tapering dose of systemic prednisone as well as topical prednisolone acetate prior to enucleation and nasal biopsy. Additionally, the absence of active inflammation does not rule out the possibility of an inactive stage of an immune-mediated disease process.

### Differential diagnosis: X-linked PRA

Multifocal depigmented lesions with early nyctalopia followed by a complete loss of vision have been described in PRA in the Tibetan Terrier [22, 23], and cone-rod dystrophy (*crd*) in miniature long-haired dachshunds [24]. The Siberian Husky has been described as having X-linked PRA with a mutation of the *RPGR* gene [25]. Reported clinical signs associated with X-linked PRA include initial nyctalopia, ophthalmoscopic signs of retinal degeneration, and diminished rod-mediated function observed at 2-4 years of age [25]. Although the age of 2 dogs and the observed nyctalopia in 1 dog (vision testing results with different light settings were not available for the other two dogs) were consistent with clinical features of X-linked PRA, normal ERG findings and a negative genetic test result in 1 dog ruled out X-linked PRA due to a *RPGR* mutation. Other types of inherited retinal degeneration, however, cannot be completely ruled out. Unfortunately, pedigree information was not available for any patient. Future studies on pedigree analysis will help investigate the genetic contribution of this disease. ERG recording with dim flashes during dark adaptation, as suggested in the first canine ERG protocol, might have supported the findings of abnormal dark adaptation in quantifiable maze tests [26]. It was not possible to run the tests due to insufficient sedation during dark adaptation.

### Differential diagnosis: nutritional deficiency

As one of the patients had been a stray, another consideration was previous nutritional deficiency. Vitamin E deficiency in particular has been reported to manifest as multifocal retinal lesions in dogs and horses and its relationship with retinal pigmented epithelial dystrophy has been suggested among several canine breeds [27–30]. Nutritional etiology is made less likely by a lack of evidence of hyperfluorescent lipofuscin accumulation on cSLO and histologic evidence of vitamin E deficiency and the incidence of 3 cases with similar signalment and clinical presentations in geographically different locations.

### Differential diagnosis: ischemia

A final possible etiology is retinal atrophy secondary to increased IOP and ischemia. In case 1, glaucoma was documented OS with chorioretinal lesions being observed OU. While intermittent subclinical IOP spikes may have contributed to noted retinal changes OD, there has been no documented IOP elevation over a 21-month follow up period. Conversely, the retinal lesions were observed OD only in the case 2 while the patient had chronic glaucoma OU. Considering OS had a small area of tapetum and relatively more RPE pigment than OD, one could hypothesize that less pigmented eyes are more susceptible to the potential ischemic damage. The wedge-shaped retinal atrophy observed with IR cSLO and the delayed choroidal filling in the FA and ICGA in case 1 are highly suggestive of ischemic lesions along watershed zones. In case 1, choroidal thinning associated to choroidal vasculature attenuation was also noted inferiorly. This was seen as a decrease in the lumen size of the choroidal vessels on OCT high-resolution cross-sections, which could further support the hypothesis of ischemia. Histopathologic evaluation confirmed choroidal thinning and atrophy in cases 1 and 2. In dogs, nonhuman primates, and humans pyramidal-shaped areas of choroidal and retinal degenerations extending from the optic nerve head consistent with watershed zones have been described with marked IOP elevations [17, 31, 32]. Anterior ischemic optic neuropathies caused by atherosclerosis and non-arteritic etiologies have also been reported to cause watershed zones in angiograms [33, 34]. Watershed zones were also observed in human patients with normotensive glaucoma and 46-90% of human subjects with no known ocular disease [35–37]. Retinal angiography is relatively infrequently performed in veterinary patients, but based on our experience with healthy research dogs without any known ocular or systemic diseases, watershed zones can also be found sporadically in normal canine eyes with FA and ICGA (data not shown). Thus, the watershed zones found in our patients do not confirm an ischemic etiology.

The pattern and dimensions of the chorioretinal lesions resemble lobules served by choroidal end arterioles which may further support ischemic etiology. A recent manuscript on decreased luminal areas of the short posterior ciliary arteries in dogs with glaucoma supports the potential ischemic etiology associated with decreased choroidal perfusion in these cases [38].

### Ellipsoid zone in OCT

The integrity of the ellipsoid zone in OCT indicates normal alignment of membranous discs in the photoreceptor outer segments, which is necessary for the normal functioning of photoreceptors. In humans, an intact subfoveal ellipsoid zone is highly correlated with visual acuity, and disrupted or absent ellipsoid zone has been reported with a number of retinal diseases [39]. While the dog in case 1 exhibited decreased visual function under dim light conditions, the ellipsoid zone at least in well-preserved retinal regions appeared to be normal.

### Summary

To summarize, we present 3 Siberian Huskies with multifocal, moderate to severe retinal atrophies with depigmented retinal lesions in the inferior fundus along with wedge-shaped areas of choroidal vessel attenuation in the superior fundus. IR cSLO, FA, and ICGA in 1 dog revealed delayed perfusion along watershed zones, which were correspondent to the area with retinal thinning in OCT. The dog had reduced night vision, but full-field ERG failed to detect any abnormality. It must be noted that the ERG, OCT and vision testing results for the normal dogs shown in this manuscript is to provide some reference to the readers, not intended to directly compare the data. Ideally, those data should be compared with age-, sex-, breed- matched dogs with similar amount of ocular pigmentation. Histology of the enucleated glaucomatous eyes in 2 dogs showed segmental partial to full thickness retinal atrophy with loss of RPE as shown in the OCT of the fellow non-glaucomatous eye in a dog. These findings are consistent with paving-stone degeneration of the retina in humans. No active signs of inflammation were observed in either globe or nasal planum sample.

While a definitive etiologic diagnosis could not be made, the authors hope that this report with extensive diagnostic data will bring our colleagues’ attention to this disease and stimulate discussions and studies with an accumulated number of patients with long-term follow-ups. We further hope that such studies will shed light on etiology, genetics, pathophysiology, and prognosis, resulting in the development of therapies.

## Data Availability

All data generated or analyzed during this study are included in this published article

## Abbreviations

crd: Cone-rod dystrophy
cSLO: Confocal scanning laser ophthalmoscopy
CSU: Colorado State University
dSLR: Digital single lens reflex
ERG: Electroretinography
FA: Fluorescein angiography
I: Inferior
ICG: Indocyanine green
ICGA: Indocyanine green angiography
IN: Inferonasal
IT: Inferotemporal
IOP: Intraocular pressure
ICA: Ridocorneal angle
MSU: Michigan State University
OD: Right eye
ONH: Optic nerve head
OS: Left eye
PACG: Primary angle-closure glaucoma
PLR: Pupillary light reflexes
PP: Peripapillary
PRA: Progressive retinal atrophy
RPGR: Retinitis pigmentosa GTPase regulator
SN: Superonasal
ST: Superotemporal
UDS: Uveodermatologic syndrome
